# Plasmablasts During Acute Dengue Infection Represent a Small Subset of a Broader Virus-specific Memory B Cell Pool

**DOI:** 10.1016/j.ebiom.2016.09.003

**Published:** 2016-09-07

**Authors:** Ramapraba Appanna, Srinivasan KG, Mei Hui Xu, Ying-Xiu Toh, Sumathy Velumani, Daniel Carbajo, Chia Yin Lee, Roland Zuest, Thavamalar Balakrishnan, Weili Xu, Bernett Lee, Michael Poidinger, Francesca Zolezzi, Yee Sin Leo, Tun Linn Thein, Cheng-I Wang, Katja Fink

**Affiliations:** a Singapore Immunology Network, A*STAR, Singapore; b Communicable Disease Centre, Institute of Infectious Disease and Epidemiology, Tan Tock Seng Hospital, Singapore; c Department of Medicine, Yong Loo Lin School of Medicine, National University of Singapore, Singapore

**Keywords:** Dengue, Plasmablasts, Memory B cells, Antibodies

## Abstract

Dengue is endemic in tropical countries worldwide and the four dengue virus serotypes often co-circulate. Infection with one serotype results in high titers of cross-reactive antibodies produced by plasmablasts, protecting temporarily against all serotypes, but impairing protective immunity in subsequent infections. To understand the development of these plasmablasts, we analyzed virus-specific B cell properties in patients during acute disease and at convalescence. Plasmablasts were unrelated to classical memory cells expanding in the blood during early recovery. We propose that only a small subset of memory B cells is activated as plasmablasts during repeat infection and that plasmablast responses are not representative of the memory B cell repertoire after dengue infection.

## Highlights

• Antibody sequences and functions were analyzed in longitudinal acute and convalescent samples from dengue patients
• Plasmablast antibodies were virus glycoprotein-specific whereas memory B cell-derived antibodies bound to more viral proteins
• plasmablasts seem to be activated from only a small subset of memory B cells

Antibody-mediated immune memory is orchestrated by various B cell types that are relevant during different phases after an infection. Antibody-secreting cells or so-called plasmablasts are generated from activated specific memory B cell a few days after re-infection. However, little in known whether the antibodies produced by these plasmablasts are relevant for protection in humans and whether the parent memory B cells are further maintained in the memory pool, possibly as affinity-matured versions of the original clones. This is important in the context of vaccination since the repertoires of individual B cell subsets could represent biomarkers to assess efficacy and long-term protection. In addition, the generation of “protective” B cell subsets could potentially be influenced by vaccine design and by the use of adjuvants.

We studied the relationship of plasmablasts and memory B cells in longitudinal blood samples from dengue patients. Dengue virus (DENV) has four serotypes and pre-existing antibodies can be cross-protective or can enhance disease after a heterologous infection via Fc-gamma-receptor-mediated uptake of virus-antibody complexes. B cell memory can therefore be both beneficial and detrimental. Here we studied plasmablasts and DENV-specific memory B cells and their relationship and protective potential by assessing antibody sequences and monoclonal antibodies. We found that both populations produced largely serotype cross-neutralizing antibodies, whereas more plasmablast antibodies were neutralizing. Few plasmablast clones could be found in the memory pool, suggesting that only a subset of memory B cells is activated during acute disease and that a separate repertoire of cells is retained as longer-term memory. In this study we started to dissect the complexity of B cell immune memory to dengue infection and the finding can inform further investigations into which immune cell subsets are disease-enhancing after a heterologous infection.

## 1 Introduction

With 390 million people infected every year, dengue is now a global concern [1]. Over the past decades, the virus has spread from South East Asia to regions across the world with climates favorable for breeding of the transmitting vector, the *Aedes* or “tiger” mosquito. In Singapore, where this study was conducted, dengue is endemic and approximately half the adult population is seropositive, providing an excellent opportunity to compare primary and secondary (memory) responses. The dengue virus (DENV) complex comprises four antigenically related viruses (DENV-1 to 4) from the flavivirus family, and infection with one serotype generates both serotype-specific and cross-reactive antibodies [2]. During heterotypic re-infection, the antibody response is dominated by cross-reactive antibodies binding to regions in the viral proteins that are conserved across all serotypes 3, 4. At the same time, neutralizing antibodies against the serotype of the previous infection are often amplified more efficiently than antibodies against the new infecting serotype, which can result in increased disease severity when an individual is re-infected with a different serotype, a phenomenon previously described as original antigenic sin 5, 6.

B cell activation, including the activation of pre-existing memory B cells (MBC), contributes to a substantial plasmablast response during acute heterologous infection 7, 8, 9, resulting in a high increase in neutralizing antibody titers [10] that contribute to temporary cross-protection against all four serotypes. Recently, we demonstrated that this plasmablast response is polyclonal, but all antibodies cloned from the genes of individual plasmablasts recognized the envelope (E) glycoprotein. In contrast, the majority of previously reported DENV-specific MBCs isolated from the blood of recovered dengue patients were specific to either prM, a membrane protein expressed on immature, non-infectious virus particles, or to non-structural proteins, notably NS1 11, 12, 13, 14, potentially indicating separate pathways of development between plasmablasts and classical MBCs.

The establishment of multiple levels of B cell memory has been suggested previously in mice. It was observed that IgM^+^ germinal center (GC) derived MBCs re-entered GC reactions upon re-infection, whereas IgG^+^ GC-derived MBCs almost exclusive differentiated into plasmablast [15]. Another elegant study in wild-type mice documented the generation of two distinct memory populations after immunization with the model antigen phycoerythrin: a long-lasting IgM memory population and a more short-lived IgG memory population. Upon re-immunization, switched memory cells differentiated into plasmablasts and proliferated to increase the memory B cell pool without further affinity maturation [16]. In contrast, the response of IgM memory B cells after re-immunization was inhibited by high amounts of specific IgG in the serum masking the antigen [16]. In B cell receptor (BCR)-transgenic mice, the formation of plasmablasts was facilitated by high affinity binding to the BCR [17] [18], a high antigen-to-B cell ratio, and a strong BCR signal 19, 20, but this system is limited in that only one epitope can be studied. During a natural viral infection, B cells respond to multiple viral epitopes, and antibodies with both high and low neutralizing capacities can have similar affinities [21]. Thus, affinity alone does not determine the efficacy of an anti-viral response, and the different biological functions of plasmablasts versus memory B cells and long-lived plasma cells post primary infection are not clear.

In humans, plasmablasts appear in the blood five to seven days after infection or vaccination. Human plasmablasts have been studied extensively to monitor vaccine- or natural infection-induced specific B cell responses and to generate disease-specific human monoclonal antibodies 8, 22, 23, 24, 25, 26. Moreover, the plasmablast response was reported to be predictive of antibody titers at least during early convalescence 22, 24. Lavinder et al. studied whether plasmablasts or MBCs contributed to the serum antibody pool after tetanus vaccination and found little repertoire overlap, concluding that only a small fraction of plasmablasts and MBCs contributed to long-lived humoral immune memory [27].

The aim of the current study was to determine the repertoires and the potential protective capacity of plasmablasts versus memory B cells in the same individuals during acute dengue disease and after recovery, and to determine the developmental relationship between these two B cell subsets.

## 2 Methods

### 2.1 Patients

The study was approved by the Institutional Review Board of Singapore National Healthcare Group Ethical Domain (DSRB B/05/013), and patients gave written informed consent. Adult patients (age > 21 y) presenting at community primary care clinics with acute-onset fever (> 38.5 °C for 72 h) without rhinitis or clinically obvious alternative diagnoses were included in the study. Whole-blood samples were collected into EDTA-vacutainer tubes (Becton Dickinson) at recruitment (acute phase), at 3–7 d (defervescence), and at 3–4 wk. after fever onset (convalescence). Patients were diagnosed by DENV-specific RT-PCR. DENV-specific IgM and IgG Abs were detected by ELISA using commercially available Panbio kits (Inverness Medical, Queensland, Australia). All twelve patients described in this study were classified as having primary or secondary infections based on DENV-specific serum IgG antibodies at time of fever onset (Table 1).

### 2.2 Single-Cell Sorting

Fresh or thawed PBMCs were labeled with antibodies against CD20, CD27, CD19 (BioLegend), CD38, and CD138 (BD Pharmingen). Dengue-specific memory B cells were identified based on their capacity to bind to Alexa Fluor 594-labeled DENV-1, Alexa Fluor 488-labeled DENV-2, and Alexa Fluor 647-labeled DENV-3. Live virus particles were labeled (Protein Labeling Kit from Molecular Probes) and inactivated with DEPC [29]. PBMCs were stained and resuspended in sorting buffer (PBS, 2% FCS, 2 mM EDTA) for sorting into 96-well PCR plates using a FACSAria (BD). The plates contained 10 mM Tris-HCL with 40 U/μl RNase inhibitor (Promega), 10 μl per well. Plates were placed on dry ice immediately after sorting and stored at − 80 °C.

### 2.3 Ig Cloning, Expression, and Purification

The mRNA of human IgG and IgM heavy and light chains was amplified from single B cells as described previously [26], using modified primer sets for RT-PCR (Table S1). One-step RT-PCR (OneStep RT-PCR Kit, Qiagen) was performed using forward primers in the heavy and light chain leader sequence and reverse primers in the constant region of IgG, IgM, kappa, or lambda. Reported primers were used for the nested IgM and IgG PCR [26] for cloning into expression vectors, RT-PCR products were used for nested IgG-PCR with modified primers (Table S1): *Sal*I and *Nhe*I sites were added at the 5′ and 3′ ends of the H chain, and *Sal*I and *Bbv*CI/*Not*I sites were added at the 5′ and 3′ end of the kappa or lambda light chains, respectively. The PCR products were cloned into the Ptt5 mammalian expression vector [30] (licensed from the National Research Council Biotechnology Research Institute, Montreal, QC, Canada). Heavy and light chain plasmids (IgG1 format) were co-transfected into the HEK293-6E cells using 293-Transfectin (Invitrogen). Transfected 293-6E cells were allowed to secrete antibodies in serum-free F17 media supplemented with 20% TN1 for 5 days. Antibodies were purified using protein G beads (GE Healthcare).

### 2.4 Ig Gene Sequencing

The PCR products of the heavy and light chain variable regions from single B cells were sequenced by Sanger sequencing. The quality of the sequences was checked with CodonCode Aligner software, sequences were trimmed and further analyzed using the IMGT database [31] (http://www.imgt.org/). For 454 sequencing, immune repertoire cDNA libraries were prepared using the Human BCR heavy chain primers (iRepertoire Inc., Huntsville, AL, USA) covering the V and C regions. In brief, 1 ng of total RNA isolated from sorted cells was reverse transcribed and amplified using Qiagen One-Step RT-PCR kit. The reaction mix for the first round of amplification contains a pool of primers targeting the V region and reverse primers that add barcodes for demultiplexing [32]. Secondary PCR was carried out to further enrich for heavy chain sequences using a Qiagen Multiplex PCR Kit and the iRepertoire® 454 Lib-A primers, according to the instructions described in iRepertoire® manual. Amplified PCR products were electrophoresed using a 2% agarose gel, and PCR fragments in the size range of 300–475 bp were gel-purified using the Qiaquick Gel Extraction Kit (Qiagen). The purified fragments were quantified using the Quant-iT ™ Picogreen® dsDNA Assay kit (Invitrogen) and submitted for sequencing (Macrogen Inc., Seoul, South Korea). Each of the patient samples were sequenced using the GS-FLX Titanium 454 platform (Roche, Mannheim, Germany) to generate 400 bp single end reads at a depth of 250,000 sequences per sample.

### 2.5 Sequence Processing and Analysis

454 sequences with a minimum length of 250 bases were uploaded to IMGT/HighV-QUEST v1.5.1. The output list was filtered by removing ‘unproductive’ and ‘unknown’ sequences. Sequences with a potential insertion/deletion, low V region identity (< 84.98%) compared to germline sequences, and sequences with missing Cys 104 were further removed before analysis of the remaining sequences in MiXCR [33]. These sequences were saved in FASTA format to use as input for MiXCR (version 1.8.2) [33]; applying presets to optimize the analysis of RNA-Seq data, MiXCR aligned the sequences to reference V, D, J and C genes of B-cell receptors; using these alignments we extracted full CDR3 sequences, discarding those backed up by just one mapped read from further analyses. For each patient and cell type, V gene usage and isotype (C gene) information was extracted from MiXCR results, selecting the best hit(s) according to alignment scores. Individual CDR3s were grouped together into clones, defined here as a group of CDR3s with the same V, D and J gene usage, with the same length and sharing at least an 85% sequence identity [34]. R (version 3.3.1) was used to perform global pairwise alignments between all the possible unique permutations of CDR3s with the same VDJ combination and length, and construct a distance matrix with 100% sequence ID as the distance measure; single linkage hierarchical clustering was applied, cutting the tree at height equal to 15% sequence dissimilarity to define the clones. Both individual CDR3 and CDR3 clone appearance across cell types within a patient was studied, and Venn diagrams were drawn (see Fig. 2A,D). A clone is considered to appear in two different cell types when at least one of the CDR3s in one cell type shares at least an 85% sequence identity with at least one CDR3 in the other cell type. Mutation analysis in Fig. 2E was done using the IMGTJunctionAnalysis tool [35].

### 2.6 Cell Lines, Virus Strains and Virus Production

Virus was produced in C6/36 mosquito cells (American Type Culture Collection). The following patient-isolate strains were precipitated using polyethyleneglycol (PEG) and were resuspended in NaCl-Tris-EDTA buffer for use in the ELISA: DENV-1 08K3126 (unpublished genotype I strain isolated by the Environmental Health Institute, Singapore), DENV2-TSV01 (AY037116.1), DENV3-VN32/96 (EU482459), and DENV4-2641Y08 (HQ875339.1).

### 2.7 Western Blot

C6/36 cells were seeded in a 75 cm^2^ flask and were infected with DENV-1 08 K3126, DENV2-TSV01, DENV3-VN32/96, or DENV4-2641Y08 at a multiplicity of infection (MOI) of 1 when they reached 70–80% confluence. Culture medium was removed 24 h after infection and cells were washed twice with cold PBS before lysis in cold RIPA Buffer containing protease inhibitors (Complete, Roche). Lysates were collected and centrifuged at 14,000 *g* for 15 min to pellet the cell debris. The supernatants containing viral proteins were collected and stored at − 20 °C. Cleared lysates were heated at 95 °C for 5 min in non-reducing Laemmli buffer prior to separation by SDS-PAGE (NuPAGE 4–12% Bis-Tris Gel: Invitrogen) and electro-transfer to PVDF membranes (Hybond-P; Amersham, GE Healthcare). The membranes were incubated with recombinant antibodies or with a pool of plasma from dengue-immune healthy donors (1:20,000), followed by peroxidase conjugated goat anti-human IgG (1:10,000; Jackson ImmunoResearch).

### 2.8 ELISA

DENV-specific ELISAs were performed by coating high-binding 96-well plates overnight with PEG-precipitated DENV serotypes 1–4 or with recombinant E proteins generated in S2 cells as described previously [10]. Plates were blocked with PBS, 0.05% Tween 20, and 3% skim milk. Supernatants from Ab-expressing HEK cells were incubated on virus-coated plates for 1 h at room temperature before washing with PBS 0.05% Tween 20 and detection with a secondary anti-human IgG-HRP (Sigma). To determine the absolute concentration of IgG, plates were coated with anti-Ig Ab (Caltag). Different concentrations of an IgG standard were included to generate a standard curve and pooled serum from dengue-immune healthy donors was used as a positive control. TMB substrate solution (Sigma) was used for all ELISAs. An OD value at least 2-fold higher than the background was defined as a positive signal.

### 2.9 Virus Neutralization Assay

U937 cells expressing DC-SIGN were seeded overnight on 96-well plates [10]. A six point dilution series of protein G–purified mAbs diluted in RPMI 1640 medium without FCS were incubated with a constant amount of DENV-1 05K2916 (EU081234), DENV-2 TSV01, DENV-3 VN32/96, or DENV-4 2641Y08 at an MOI of 1 for 30 min at 37 °C. Plasma or mAb–virus mixtures were then transferred onto the U937-DC-SIGN cells and incubated for 2 h at 37 °C before adding RPMI 1640, 10% FCS. After incubation overnight, cells were stained intracellularly with Abs against NS1 and E protein and analyzed on a FACSVerse cytometer (Becton Dickinson). Data were analyzed using FlowJo software (TreeStar). The proportions of infected cells were plotted against the dilution factor, and the EC50 was calculated with Prism5 (GraphPad Software), applying a three-parameter non-linear curve fit.

### 2.10 Immunohistochemistry

BHK-21 cells were seeded in 96-well plates and infected with DENV-1 08K3126, DENV-2 TSV01, DENV-3 VN 32/96, or DENV-4 2641Y08. After 48 h, the cells were fixed with 4% paraformaldehyde for 20 min at room temperature. For intracellular staining, cells were permeabilized with PBS/0.1% Triton X-100 for 15 min at room temperature. Cells were then washed and blocked with 1% BSA in PBS for 2 h at room temperature. Fixed cells were incubated with primary antibodies at a concentration of 1 μg/ml at room temperature for 1 h, followed by Alexa Fluor 488 goat anti-human IgG (Invitrogen) 1:2000 at room temperature for 1 h. Nuclei were counterstained with Hoechst (Invitrogen) for 5 min (1:15,000 in PBS). Stained cells were visualized using a confocal microscope (OLYMPUS).

## 3 Results

### 3.1 Antibody Variable Region Gene Usage Differs between Plasmablasts and Memory B Cell from Longitudinal DENV Patients Samples

**Fig. 1:**
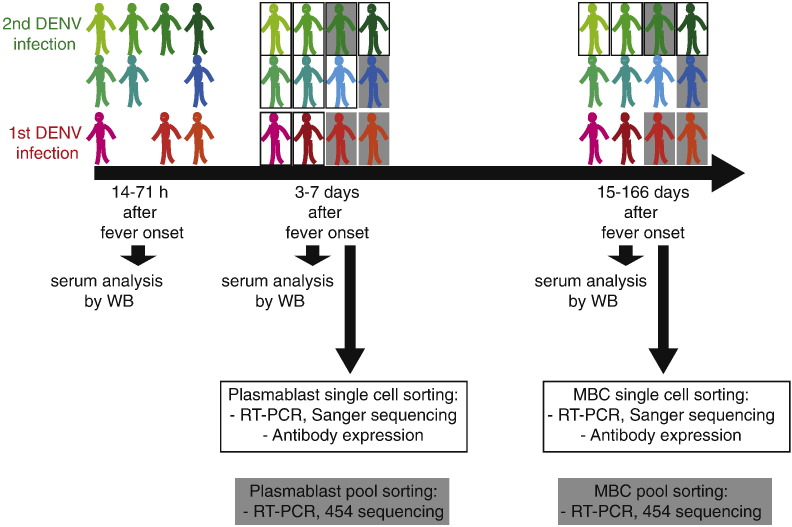
Study setup and time points of sample collection. Dengue patients with a primary or secondary infection were enrolled into the study between 14 and 71 h after onset of fever. The same patients were recalled 4–7 days and 15–166 days after onset of fever. Each patient is color coded. Samples from patients with a black box were used for single B cell sorting, sequencing and Ab expression. Samples from patients with a grey-shaded box were used for pooled B cell sorting and 454 sequencing.

**Fig. 2:**
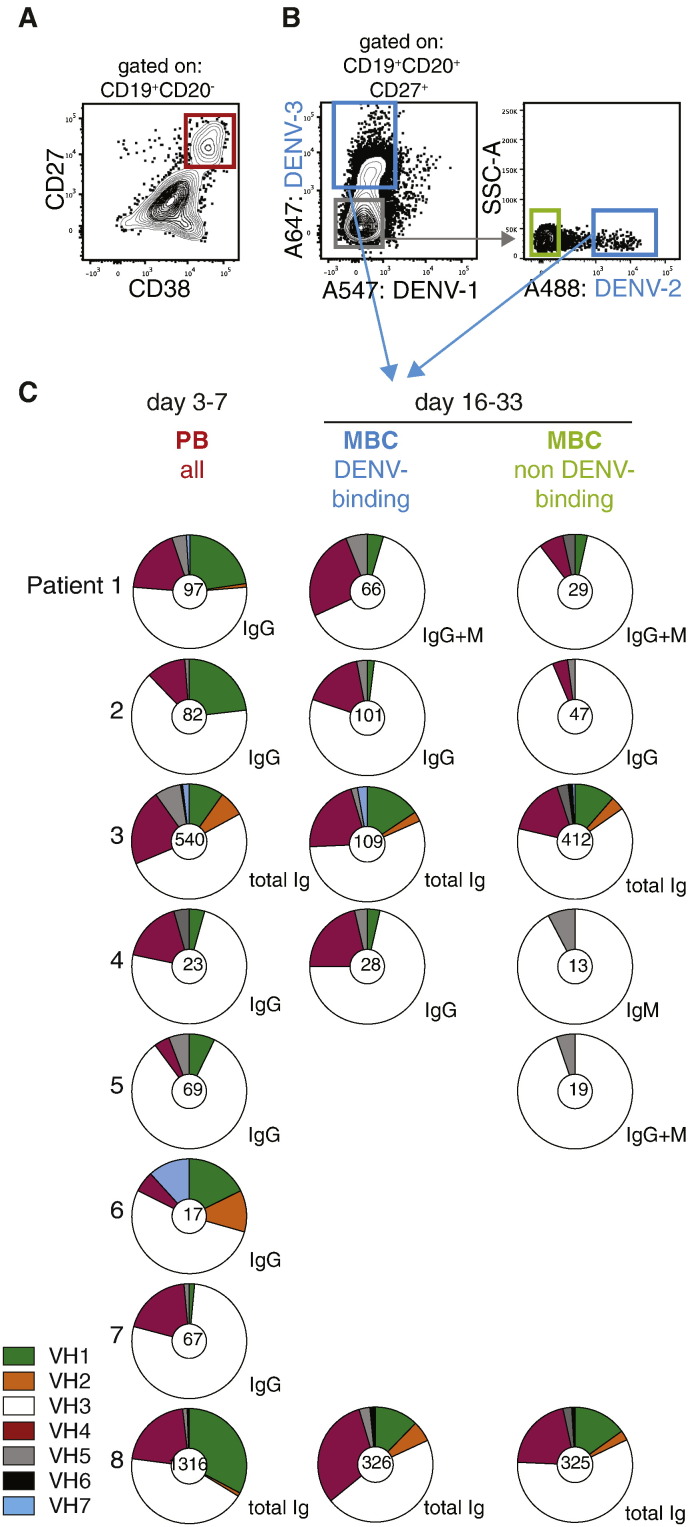
B cell repertoires change between acute phase and early convalescence. A and B) Flow cytometry plots representative of a dengue patient (Patient 3) to illustrate the sorting strategy. A) Sorting of CD19^+^, CD20^−^, CD27^hi^, CD38^hi^ plasmablasts. B) Sorting of CD19^+^, CD20^+^, CD27^+^ DENV-binding memory B cells (MBC) (indicated in blue squares) and non-DENV-binding MBCs (indicated in green square). Sequences from DENV-2 and DENV-3-binding MBCs were pooled as “DENV-binding MBCs”. **C**) VH gene family usage of plasmablasts (PB), DENV-binding MBCs, and non DENV-binding MBCs. The total numbers of unique CDR3 sequences per sample is indicated in the center of pie charts. Analysis included IgG sequences, IgM sequences or total sequences, as indicated next to the charts. Cells from patients 3 and 8 were analyzed with 454 sequencing; for all other patients, single B cells were analyzed by Sanger sequencing. A Wilcoxon matched-pairs signed rank test showed a statistically significant difference only for VH3 and VH4 usage between PB and MBC-neg cells (*p* = 0.03).

To study the evolution of the B cell repertoire during DENV infection, blood samples from twelve patients infected with DENV virus serotype 2 (eleven patients) or serotype 3 (one patient) were collected during acute disease and after recovery. The study time points and analysis performed are summarized in Fig. 1. In a primary infection, DENV-specific IgG can only be detected five to seven days after fever onset. Therefore, to distinguish between patients with pre-existing DENV immunity and those without previous exposure to the virus, DENV-specific IgG antibodies were measured by ELISA within 72 h of fever onset. This analysis identified eight patients with secondary infections and four patients with primary infections (Table 1). Plasmablasts (PB; CD19^+^ CD20^−^ CD27^high^ CD38^high^ cells) were sorted from peripheral blood of patients with secondary infection three to seven days after fever onset (see Fig.2A and Supplementary Fig.1A online for detailed sorting strategy). Specific and non-specific memory B cells (MBCs; CD19^+^ CD20^+^ CD27^+^ CD38^low^ cells) were sorted 16 to 25 days after fever onset using fluorescently labeled virus to distinguish between DENV-binding and non-binding cells (DENV^+ or –^ cells), respectively (Fig. 2B and Supplementary Fig. 1B online). The DENV serotypes used for MBC sorting are listed in Table S2. After analyzing the first two patients we noticed that almost all MBCs were serotype cross-reactive regardless of the virus used for sorting. For the subsequent secondary patients we used the DENV-3 probe, which showed the highest % of binding cells. Very few MBCs bound to DENV-1 and this population was therefore not further analyzed. Across all patients analyzed in this study 0.1–19% of total lymphocytes were activated as plasmablasts and 0.5–8.1% of CD19^+^ CD20^+^ B cells bound to DENV (Table S3). Cells were sorted into 96-well plates for PCR-amplification of single cell heavy chain variable region (IgVH) and light chain variable region (IgVL) transcripts, with the exception of cells from patients 8, 11 and 12, which were analyzed by 454 sequencing [32]. For patient 3, both single cell and 454 sequencing were carried out (Fig. 1). To address potential repertoire differences we compared IgVH V and J gene usage in plasmablasts, DENV-binding and non-binding MBCs in patients with secondary infections (Fig. 2C). VH usage of plasmablasts compared to DENV-binding MBCs was not significantly different, possibly due to the limited number of sequences analyzed per patient. There was a significant difference between VH3 and VH4 usage in plasmablasts compared to non-binding MBCs (*p* = 0.03). The primers used for single cell PCR amplification and for deep sequencing bind in the constant region, preserving information about variable region and isotype (Fig.2C). There seemed to be more pronounced differences in VH usage between plasmablasts and DENV-binding MBCs when the IgG-, IgM- and IgA-expressing cells were analyzed separately (Supplementary Fig. 2A). However, only the two secondary patients analyzed with 454 sequencing had sufficient numbers of sequences per isotype for a separate analysis and a statistical test could not be done. Higher VH1 gene usage in plasmablasts compared with MBCs, as we reported previously [8], was only observed in three out of five patients here, indicating that high VH1 usage in plasmablasts is not universal in DENV infection. Typically, infections such as influenza results in a large clonal expansion of individual B cells 36, 37, however only patient 6 displayed a large clonal expansion, whereas the remaining donors showed polyclonal activation and little or no clonal expansion in the plasmablast population (Supplementary Fig. 2B online and [8]). The expanded clone in patient 6 was an IgG clone and the respective antibodies bound specifically to EDIII of DENV-1, even though the patient was infected with DENV-2. This might be an exceptional case for which we don't have an explanation yet.

### 3.2 No Evidence that Acute Disease B Cell Sequences Are clonally Expanded in the Memory Pool

**Fig. 3:**
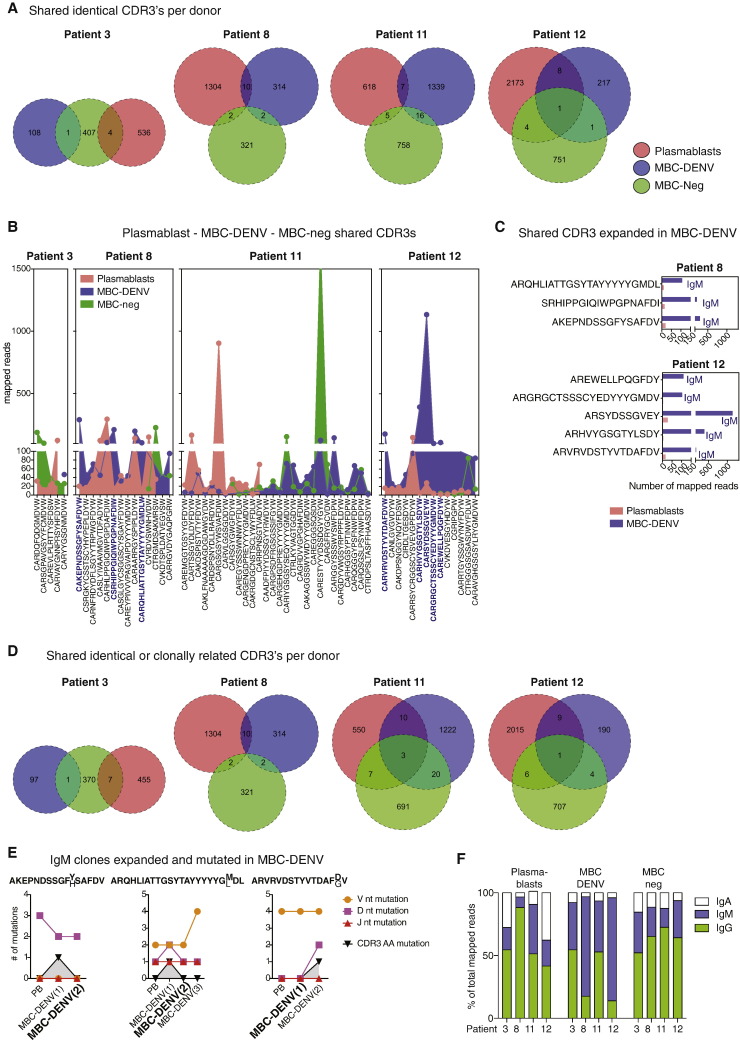
Few shared sequences between plasmablasts and memory B cells. A) Identical unique CDR3 Sequences shared between plasmablasts (PB), DENV-2-binding MBCs (MBC-DENV) and non-binding MBCs (MBC-neg) for two patients with secondary DENV-2 infection (E1291, E1392) and two patients with primary DENV-2 infection E1414, E1465. B) Numbers of mapped reads for sequences shared between PBs MBC-DENV and MBC-neg. C) number of mapped reads and isotype for PB and MBC-DENV shared sequences that are at least 10 × expanded in the memory compartment. D) Shared clones based on at least 85% identity in the CDR3 amino acid sequence. E) CDR3 mutation analysis for the three clones that were expanded and mutated in the MBC-DENV repertoire. The MBC-DENV CDR3 sequence in bold in the x-axis represents the expanded clone according to the number of mapped reads. The CDR3 amino acid sequence and mutation is indicated above the graphs, with bold characters indicating the dominant sequence. F) Isotype distribution of PB, MBC-DENV and MBC-neg sequences. Total mapped reads was used for the analysis.

Since VH and JH gene family usage seemed to differ between acute and convalescent time points (Fig. 2C and Supplementary Fig. 2A) we tested whether plasmablast and MBCs were clonally related. Pools of longitudinal plasmablasts and memory B cell repertoires analyzed by 454 sequencing were used for this comparison (Fig. 3). We found that only few CDR3 sequences were shared between plasmablasts and early convalescence DENV-2 or − 3 binding memory B cells from the same individual infected with DENV-2 (Fig. 3A). In addition, no plasmablast sequences were found in the DENV-3 binding MBC repertoire that was isolated and deep sequenced one year after infection for patient 2 who was infected with DENV-3 (data not shown).

Since repertoires during secondary infection are biased towards cross-reactive and affinity- matured cells we also included two patients with primary infection in the analysis (patients 11 and 12) but found similarly low numbers of CDR3s shared between plasmablasts and dengue-binding MBCs (MBC-DENV) (Fig. 3A). If B cell clones that participate in the plasmablast response are also retained in the memory pool we would expect a specific expansion of the respective MBC-DENV clones. Overall, 61 CDR3s were found in more than one subset and were either expanded in PB or the MBC pools (Fig. 3B). Eight CDR3 sequences from two out of four patients were at least ten times expanded (in terms of mapped reads) in the DENV-specific memory compartment compared to the plasmablast compartment (Fig. 3C). To account for affinity maturation between acute and early convalescence phase we next collapsed CDR3 amino acid sequences that were at least 85% identical into clones for each cell population [38]. Similar to the analysis of identical CDR3 sequences, few related CDR3 clones were found in both plasmablasts and MBC-DENV in the same individual (Fig. 3D). This was true both in the case where the DENV serotype of the recent infection or a heterologous serotype was used for MBC sorting (Supplementary Table S1). Only three out of eight shared clones were mutated in MBC-DENV compared to plasmablasts but the amino acid mutations were found in the non-expanded MBC-DENV CDR3s (Fig. 3E). Surprisingly, the MBC-DENV expanded clones contained exclusively IgM sequences (Fig. 3C and E). In fact, a large fraction of DENV-specific memory B cells expressed IgM, compared to plasmablasts, which were dominated by IgG-expressing cells in both primary and secondary infection cases and compared to non-DENV binding MBCs, which predominantly expressed IgG (Fig. 2F). We assume that a significant number of B cells in the memory IgM repertoire bind to DENV with relatively low affinity and that these cells were enriched during the MBC sorting. In fact, we also observed DENV binding in two non-dengue immune individuals (Fig. S1C).

Overall, the majority of clones activated during the acute phase in the plasmablast response were not represented in the circulating MBC repertoire at convalescence and there was no evidence of specific expansion or affinity maturation for the related MBC-DENV clones.

### 3.3 Plasmablast-Derived mAbs Are E Protein-Specific whereas MBC-Derived mAbs Bind to a Wider Range of Viral Antigens

We next tested whether the different VDJ gene usage of plasmablasts and memory B cells resulted in different B cell specificities. We previously reported that plasmablasts were predominantly E protein-specific, but did not analyze the specificity of the memory B cells in that study [8]. Others have shown that more than half of DENV-specific memory B cells were specific to prM and to non-structural (NS) proteins 11, 12, 13, 14. We first analyzed the specificity of Abs in plasma collected from patients within 72 h of fever onset, during acute disease (day 3–8), and at convalescence (day 15–166) by western blot (Table 1). Antibodies to prM, E, were detectable during acute disease in six out of seven patients with a secondary infection, whereas NS1-specific Abs were mostly observed during convalescence. Hence, prM -specific antibody-secreting cells were generated or mobilized during acute disease, but they did not seem to participate in the plasmablast response, which only contained E-specific mAbs, consistent with our previous observation [8].

**Fig. 4:**
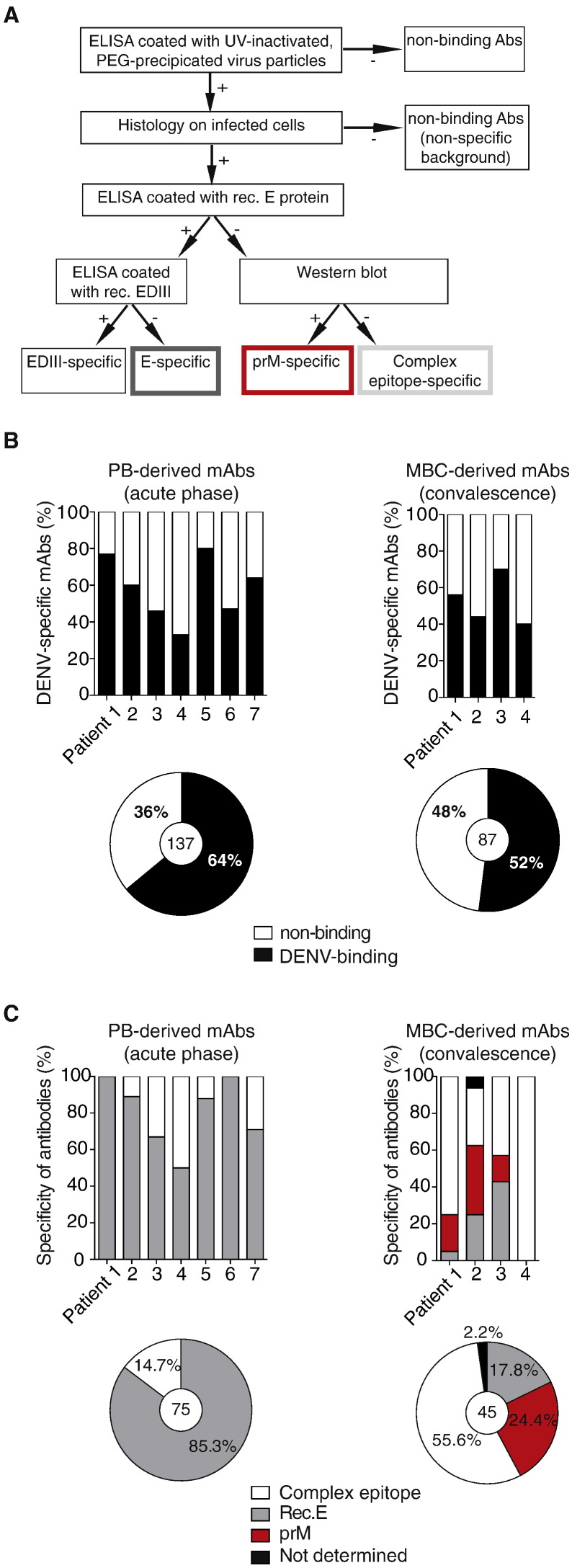
Specificity of antibodies derived from plasmablasts and memory B cells after secondary infection is markedly different. A) Overview of the assays used to assess the specificity of single B cell-derived monoclonal Abs. Abs were characterized into four groups: EDIII-specific, E-specific, prM-specific, and complex epitope specific antibodies. B) DENV-binding Abs as fractions of all Abs tested per patient and per cell type. The average of DENV-binding Abs across all patients is illustrated for PB-derived Abs and (MBC)-derived antibodies, with the total number of Abs tested indicated in the center of the pie charts. C) Percentages of Abs binding to complex epitopes, recombinant E protein (Rec. E), or prM were defined for plasmablasts (PB) from patients 1 to 7. All E-specific Abs of patient 6 bound to EDIII, whereas no EDIII-specific antibodies were observed for all other patients and time points analyzed. Binding specificity was defined for memory B cells (MBCs) from patients 1 to 4. One MBC-derived Ab could not be characterized (black bar in Fig. 2C, due to low expression levels (≤ 0.1 μg/ml).

To further explore B cell population-associated differences in specificity, recombinant monoclonal antibodies (mAb) were cloned and expressed from the IgG-expressing plasmablasts and -MBCs, isolated longitudinally from individuals during acute disease and at convalescence. Specificity of the mAbs to E protein, E domain III (EDIII, the binding site of potent neutralizing antibodies to E protein), and to prM, was determined as described in Fig. 4A. On average, 64% of the mAbs cloned from plasmablasts and 52% of the mAbs cloned from sorted MBCs bound to DENV (Fig. 4B). Of the DENV-specific mAbs derived from plasmablasts (75 unique sequences from 7 patients), 85.3% recognized recombinant E protein compared with only 17.8% of the DENV-specific mAb derived from MBCs (45 unique sequences from 4 patients), (Fig. 4C), confirming again the dominance of E protein-specific Abs during acute infection. Antibodies to prM were not detected in the plasmablasts, but accounted for 24.4% of those in the MBC population (Fig. 4C). Antibodies that bound intact virus particles in ELISA or from infected cell lines, as determined by immunohistochemistry, but did not recognize E or PrM proteins were designated as complex epitope-specific mAbs. 14.7% of plasmablast-derived mAbs compared with 55.6% of MBC-derived mAbs (Fig. 4C).

**Fig. 5:**
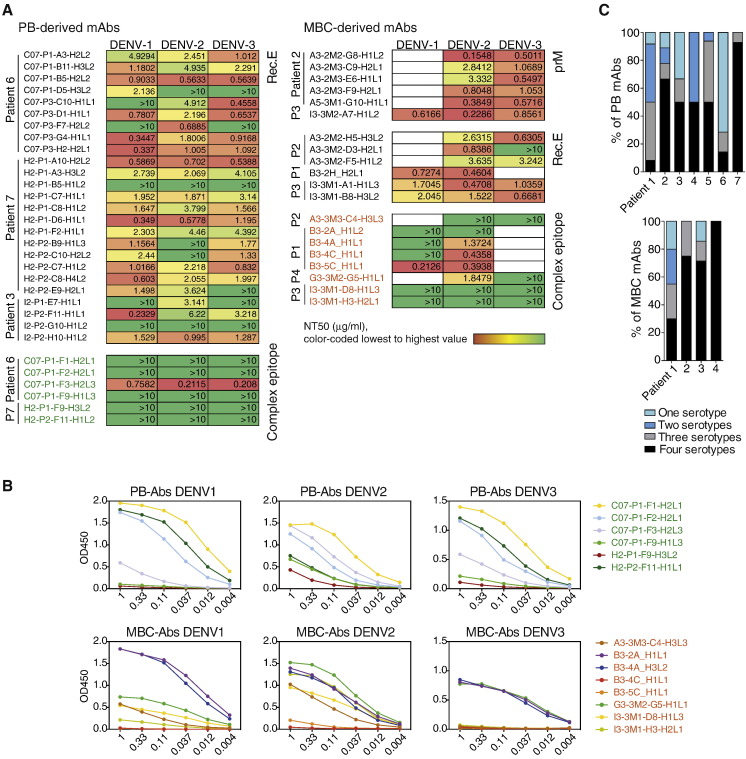
E-specific antibodies have a higher neutralizing capacity compared to complex-epitope-specific antibodies. A) Neutralizing capacity of 51 mAbs representing the different antibody groups for plasmablasts and MBCs. The patient origin and mAb ID is indicated on the left. The NT50 for PB-derived mAbs was determined for DENV-1,-2 and -3. DENV-4 was not tested since this serotype is very rare in Singapore. NT50 for the current and the previous serotype of infection (based on the neutralizing profile of the patient plasma) were tested for MBC-derived mAbs. Each NT50 value represents the mean from three independent experiments. B) Virus particle (UV-DENV) ELISA for complex-epitope-specific mAbs. C) Cross-reactivity of antibodies per patient and cell type, assessed by UV-inactivated PEG precipitated virus particle ELISA, rec.E ELISA, immunohistochemistry and western blot assays for DENV-1 to -4. Abs were grouped based on their ability to bind across all four, three, two or one serotype(s) of DENV in at least one or multiple assays.

Recent studies have suggested that Abs binding to intact virus particles but not to recombinant E protein, i.e., the complex epitope-specific mAbs described here, can be potent neutralizers 2, 12, 39, 40, 41, 42. To test the relative neutralizing capacities of complex epitope-specific mAbs and E protein-specific Abs, we compared their 50% neutralizing titer (NT50) using human U937 monocytic cells stably transfected with the DENV binding receptor DC-SIGN [2]. E protein-specific mAbs were capable of neutralizing the three serotypes tested at lower concentrations (0.1–10 μg/ml) compared with the complex epitope-specific mAbs, which typically required concentrations of > 10 μg/ml in both plasmablast and MBC populations, an amount that is considered weakly neutralizing for a DENV-specific antibody 11, 40. In patients P1 and P4, MBC-derived complex epitope-specific mAbs showed a higher serotype-specific neutralizing capacity for DENV-2, the serotype responsible for the active infection (Fig. 5A), despite similar binding to UV-treated DENV-1 and/or DENV-3 by ELISA (Fig. 5B). Very potent neutralizing antibodies are usually serotype-specific 2, 12, 39, 40, 41, 42. The antibodies tested here were, however, mostly serotype cross-reactive (Fig. 5C), representing a different yet apparently abundant class of antibodies.

Thus, cross-reactive plasmablasts present during acute disease secrete neutralizing *E*-specific antibodies, which most likely play an essential role in controlling early viral replication. Cross-reactive MBC-derived antibodies predominantly recognize complex epitopes, which are less potent functionally, but still capable of neutralizing the active infection.

### 3.4 Distinct Ig Heavy Chain Gene Usage and CDR3 Between Antibodies Isolated From DENV-specific Memory B Cells and Plasmablasts

**Fig. 6:**
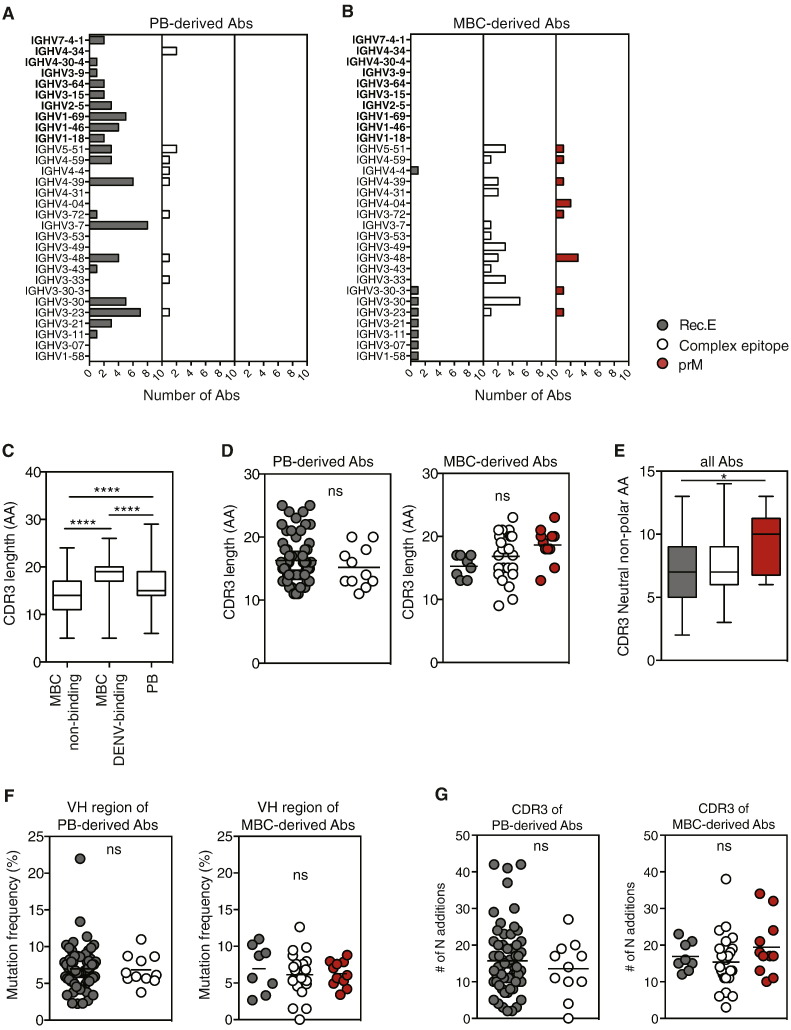
DENV-binding antibodies derived from PB and memory B cells use distinct VH genes but show similar mutation rates. A–B) VH gene family usage for epitope-specific antibodies derived from plasmablasts (PB) (A) and memory B cells (MBCs) (B). Ab specificity is indicated with different colors, and the numbers of Abs per gene family is indicated on the y axes. Significant differences between PB and MBCs were calculated with a Fisher's Exact test: prM: *p* = 1.00, Complex epitope: *p* = 0.298, Rec.E: *p* = 0.022. C) Comparison of the CDR3 length of all plasmablasts (primary and secondary infection; *n* = 603) with the CDR3 length of DENV-binding MBCs (*n* = 245) and DENV-non-binding MBCs (*n* = 163). *P* < 0.0001 (one-way ANOVA with Tukey's multiple comparison test). D) CDR3 lengths of plasmablast (PB) and MBC-derived Abs across Ab specificities. E) Content of neutral non-polar amino acids (AA) in CDR3 sequences from prM-specific Abs compared to CDR3 sequences from rec. E-specific Abs. F) VH nucleotide mutation frequencies of plasmablast-derived Abs and MBC-derived Abs of different specificity. A one-way ANOVA analysis comparing all five groups of Abs (both plasmablast and MBC-derived) showed no significant differences between plasmablasts and MBCs. G) Number of CDR3 nucleotide (N) additions for all Ab groups. For scatter plots each dot represents one Ab and horizontal lines indicate the means. Whiskers in the box and whisker graphs indicate min to max values.

Because we observed distinct non-overlapping repertoires in virus-specific plasmablasts and MBCs generated after DENV infection, we investigated whether monoclonal antibodies derived from each of the two populations could be identified and discriminated based on their Ig heavy chain variable region (IgVH) sequences. Plasmablast-derived mAbs showed a broad usage of VH1 to VH7 genes with the exception of VH6, indicating that E glycoprotein specificity was not restricted to certain VH families (Fig. 6A). For MBC-derived Abs, VH3 and JH4 usage was more dominant, typical of the naïve and MBC repertoires of normal healthy individuals [43]. Interestingly, some VH gene families with self-antigen binding potential, including VH1–69 and VH4–34 44, 45, 46, were observed specifically amongst plasmablast-derived DENV-specific Abs but not amongst MBC-derived DENV-specific Abs (Fig. 6A and B).

The length and flexibility of the CDR3 can also contribute significantly to antigen binding. We compared the IgH CDR3 length of non-DENV-binding and DENV-binding MBC- and plasmablast-derived antibodies (sorting strategy in Fig. 1A) in all cells for which a sequence was available. On average, DENV-binding MBC-derived antibodies had significantly longer CDR3 than DENV-binding plasmablast-derived antibodies and, to a greater extent, non-DENV binding MBC-derived antibodies (Fig. 6C). Analysis of epitope specificity indicated that complex epitope-specific and prM-specific antibodies seemed to account for the longer CDR3 sequences of DENV-specific MBCs compared to plasmablasts and non-DENV-specific MBCs (Fig. 6D). CDR3s of prM-specific antibodies also contained significantly more neutral non-polar amino acids than *E*-specific Abs (Fig. 6E).

The immediate appearance of plasmablasts following infection indicates that they are unlikely to participate in a germinal center reaction before their differentiation into antibody-secreting cells. We hypothesized that MBCs therefore underwent a (primary or secondary) GC reaction before their appearance in the blood during early convalescence. However, both memory B cells and plasmablasts showed similar levels of nucleotide mutations in the heavy chain variable region (Fig. 6F) and in the CDR3 (data not shown) compared to reference germline sequences (the international ImMunoGeneTics information system® http://www.imgt.org), and also showed similar levels of nucleotide additions (Fig. 4G). Similarly, a comparison of mutations in frame 1, 2, and 3 versus CDR1 and CDR2 showed that all Abs, independent of specificity and plasmablast or MBC origin, had comparable numbers of silent versus replacement mutations (Supplementary Fig. 3 online).

In conclusion, plasmablasts and DENV-specific MBCs differed significantly in their IgVH gene usage and CDR3 length, but not in their mutation rates. Therefore, despite being clonally unrelated, both populations appear to have undergone a similar level of affinity maturation and selection.

## 4 Discussion

Immune memory allows for the efficient activation and expansion of B cells during recall responses. It has long been established that a recall B cell response involves rapid generation of plasmablasts and a temporally delayed formation of germinal centers. It is less clear, however, which B cells enter each path, and how the plasmablast and classical memory B cell population is related during acute stages of infection. We find here that plasmablast and memory B cell formation after DENV re-infection involves clonally distinct B cells. The clonally related sequences were all of the IgM isotype and could represent B cells that bound to DENV with low affinity and that were enriched during the sorting with fluorescently labeled virus. This binding could be cognate or via heparan sulfate, which is a receptor for DENV. Although DENV-binding cells were sorted from the CD27^+^ memory B cell population (Fig. S1) we cannot exclude that few naïve CD27^−^ naïve B cells were also included in the memory gate. The accumulation of IgM^+^ cells in the DENV-binding but not the control memory B cell pool (Fig. 2E) further points to an enrichment of low affinity IgM B cells. We did not have a pentameric IgM expression system and were not able to verify the binding of the MBC-DENV–derived IgM antibodies. A role of IgM MBCs in maintaining memory over prolonged periods of time and the capacity of IgM MBCs to re-enter germinal centers is intriguing 15, 16 but will have to be studied in more detail in the context of dengue.

Of note, 40–60% of the cloned mABs from sorted IgG MBC-DENV cells were found to specifically bind to DENV (Fig. 4B) and this validated our sorting strategy. None of the IgG MBC CDR3 sequences, however, were shared with the PB repertoire.

We observed that memory B cell-derived, complex epitope-specific antibodies were less neutralizing than plasmablast-derived, E-specific Abs. This was surprising because a number of MBC-derived complex-specific antibodies are described in the literature to be potent neutralizers 2, 41, 42, 47. We assume that the potent neutralizers previously described, which were all serotype-specific, are rare in the memory pool and our sampling size was not large enough to pick them up. In our experiments, the majority of the DENV-specific isolated MBCs were cross-reactive, but weakly neutralizing. The sorting strategy focusing on DENV-3-binding B cells likely created a bias towards cross-reactive B cells. Nevertheless, some cross-reactive MBCs did show a higher neutralizing capacity for the recent infecting DENV serotype (DENV-2 for patients P1 and P4 with DENV-2-specific neutralization, Fig.4A). Although having a low neutralizing capacity, the large proportion of MBCs that express BCRs specific for virus particles could play an important role as antigen-presenting cells during acute viral infection.

It is curious that only E-protein specific, mostly IgG^+^ MBCs seemed to participate in the plasmablast response during re-infection while serum levels of prM and NS1-specific Abs also increased during acute disease (Table 1). PrM and NS1-specific MBCs might differentiate into antibody-secreting cells upon infection without circulating in the blood as plasmablasts. MBCs might also become long-lived plasma cells that are retained in the bone marrow and in other secondary lymphoid organs [48], accounting for the pre-existing antibodies early after fever onset (Table 1).

The dynamics of immune memory re-activation is of special interest for dengue virus infection where original antigenic sin is a prominent phenomenon that complicates efficacious vaccination against all four serotypes. In this context, it is important to understand the origin of rapidly responding plasmablasts as the effector cells of antibody-mediated original antigenic sin. Determining the particular MBC subset [28] that they are derived from is complicated because the gene expression profile and surface marker expression of MBCs change upon differentiation into plasmablasts. While our data do not solve the question about the origin of plasmablasts during acute dengue infection our findings have two main implications: a) After vaccination, plasmablasts and MBCs could be analyzed and differentiated based on their distinct repertoires, providing a potential biomarker to compare the responses between vaccine-induced and natural infections and to analyze responses to new vaccine candidates. B) The plasmablast response and the ensuing temporary burst of E-protein-specific antibodies in the plasma may not be a useful readout for long-term immune protection in dengue as it is only representative of a small fraction of the total memory B cell pool.

Memory B cell-derived antibodies isolated here and those reported previously by others 11, 12, 14 bind to E, prM, NS1 and this spectrum is more in line with the specificity serum antibodies that are produced after re-infection and during early convalescence (Table 1). Whether the re-activation of these memory B cells as antibody-secreting cells or as antigen-presenting cells, however, is crucial for protection in dengue patients remains to be studied. Analysis of both the acute phase plasmablasts and memory B cells will be crucial to start to understand the biological relevance of different levels of B cell memory that are generated after dengue infection.

## Funding Sources

The study was funded by the Agency for Science, Technology and Research A*STAR, and by the National Medical Research Council, Singapore: NMRC/TCR/005 (STOP Dengue Translational and Clinical Research (TCR) flagship grant). The funders had no role in study design, data collection and analysis, decision to publish, or preparation of the manuscript.

## Conflicts of Interest

The authors declare no conflict of interest.

## Author Contributions

RA, SKG, MHX, YXT, SV, CYL,TB and WX performed experiments. RZ established reagents used in the study. RA, SKG, DC, BL and KF analyzed data. YSL and TLT managed the patient cohort, provided patient samples and clinical data. KF, CIW designed the study, with important input from MP and FZ. KF and RA prepared graphs and figures and wrote the manuscript.

## Figures and Tables

**Table 1:** DENV-specific IgG in the plasma of patients detected by Western blot.

				Early		Acute		Convalescent	
Patient number	Patient ID	Infection	Current serotype of infection	Time	WB	Time	WB	Time	WB
1	10/63'	Secondary	DENV-2	45 h	–	4d	–	19d	–
2	10/50'	Secondary	DENV-3	17 h	–	6d	Env, prM	16d	Env, prM, NS1
3	E1392	Secondary	DENV-2	57 h	Env	3d	Env, prM	22d	Env, prM, NS1
4	E1183	Secondary	DENV-2	69 h	Env	4d	Env, prM	24d	Env, prM, NS1
5	E1407	Secondary	DENV-2	55 h	Env, prM	3d	Env, prM	16d	Env, prM, NS1
6	E1311	Secondary*	DENV-2	14 h	nd	7d	nd	15d	nd
7	C07	Secondary	DENV-2	–	–	6d	Env, prM, NS1	128d	Env, prM, NS1
8	E1291	Secondary	DENV-2	71 h	–	4d	Env, prM	23d	Env, prM, NS1
9	E1385	Primary	DENV-2	16 h	–	6d	nd	16d	Env, prM
10	C01	Primary	DENV-2	–	–	5d	nd	166d	Env, NS1
11	E1414	Primary	DENV-2	68 h	–	4d	Env, prM	17d	Env, prM, NS1
12	E1465	Primary	DENV-2	67 h	–	8d	nd	33d	nd

(–) Serum not available.

(nd) Not detected with 1:1000 dilution of plasma.

(') 10/63 and 10/50 have been described before in Ref. [8].

(*) This patient had no pre-existing DENV-binding IgG and was classified as secondary due to the highly EDIII-specific response of the plasmablasts.

## Appendix A Supplementary Data

Supplementary figures 1-3. Image 1

Supplementary tables 1-3. Image 2
