# Supplementary material for: Plasmablasts During Acute Dengue Infection Represent a Small Subset of a Broader Virus-specific Memory B Cell Pool
Source: eBioMedicine. 2016 Sep 7;12:178–88. doi: 10.1016/j.ebiom.2016.09.003 (PMC5078588; doi:10.1016/j.ebiom.2016.09.003)
Supplement: Supplementary file 1 — Supplementary figures 1-3. [file mmc1.pdf]

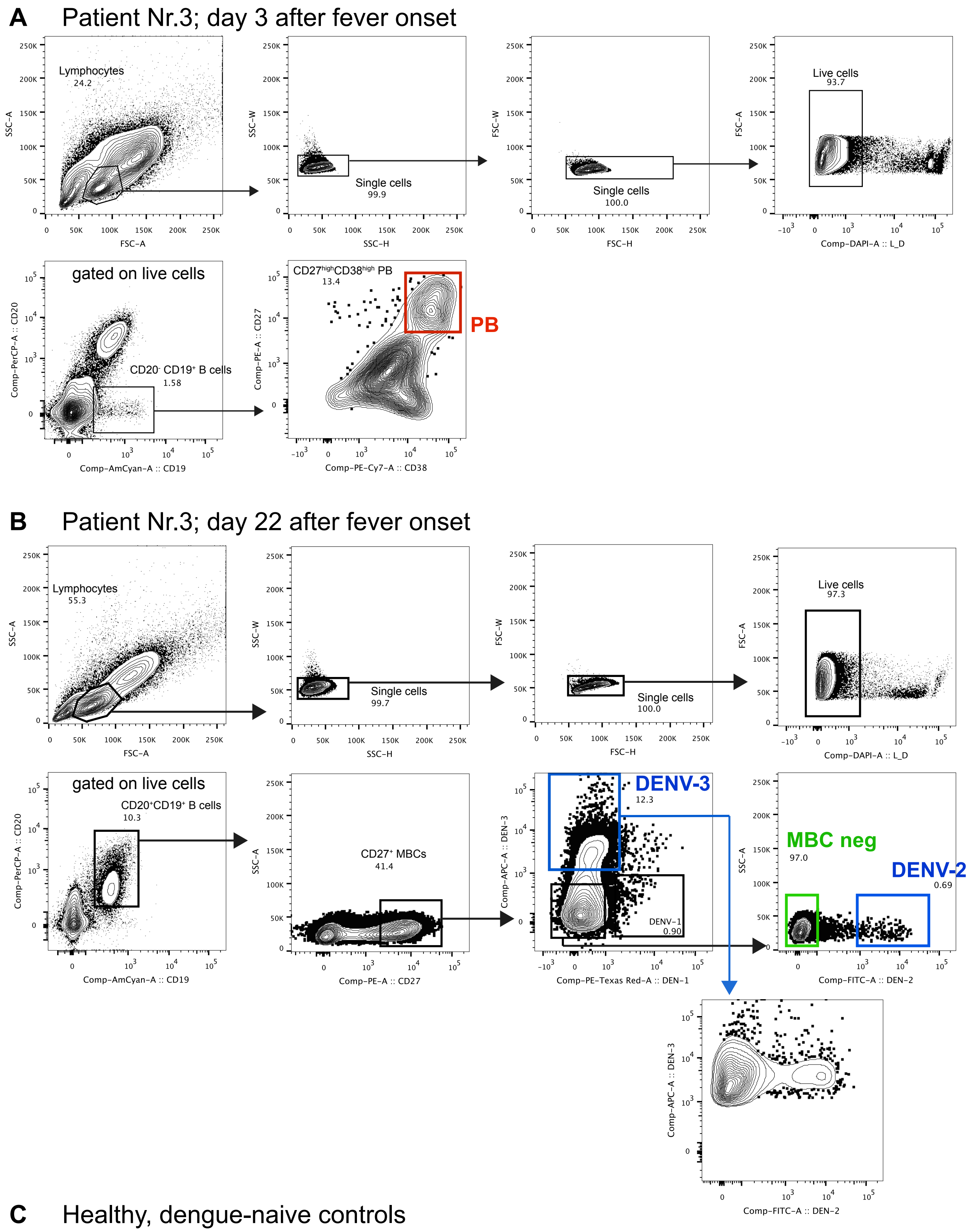

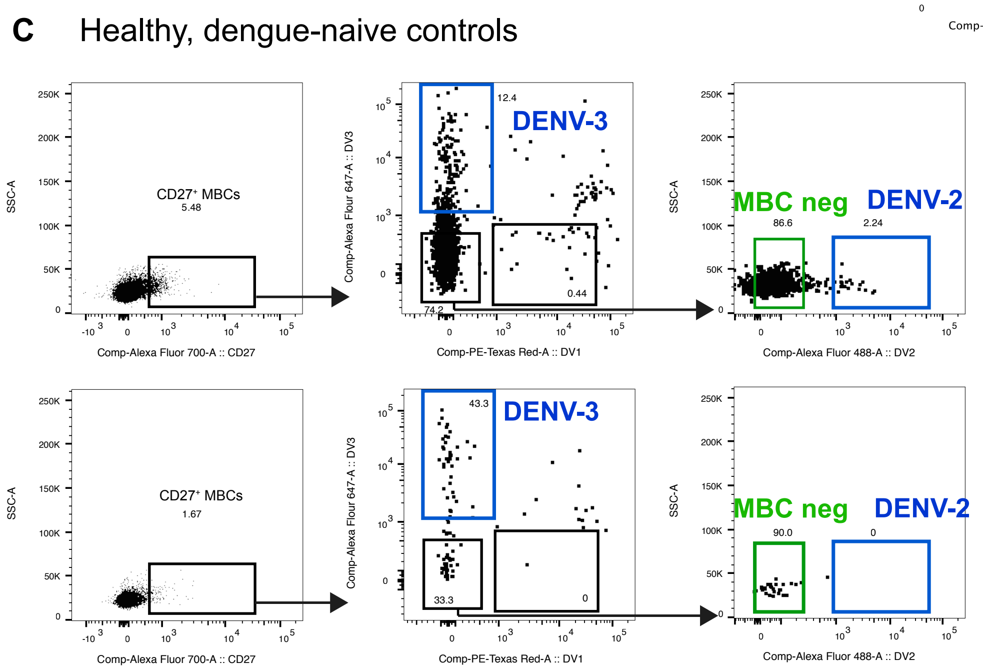

Supplementary Figure 1: B cell sorting strategy for patient samples, illustrated for patient 3. A) PBs (red box) were sorted during the acute phase of infection. B) During convalescence, DENV-3 and DENV-2 specific MBCs were sorted (blue boxes). MBCs that did not bind fluorescent DENV-1-Alexa586, DENV-2-Alexa488 and DENV-3-Alexa647 were sorted as non-DENV binding MBCs (MBC neg; green box). Since most of the eventually cloned mAbs were cross-reactive the serotype cross-reactivity of DENV-3-binding cells with DENV-2 is illustrated in a separate graph (DENV-3 vs. DENV-2). C) Two healthy, dengue-seronegative control samples were stained with the fluorescnet probes shown in (B).

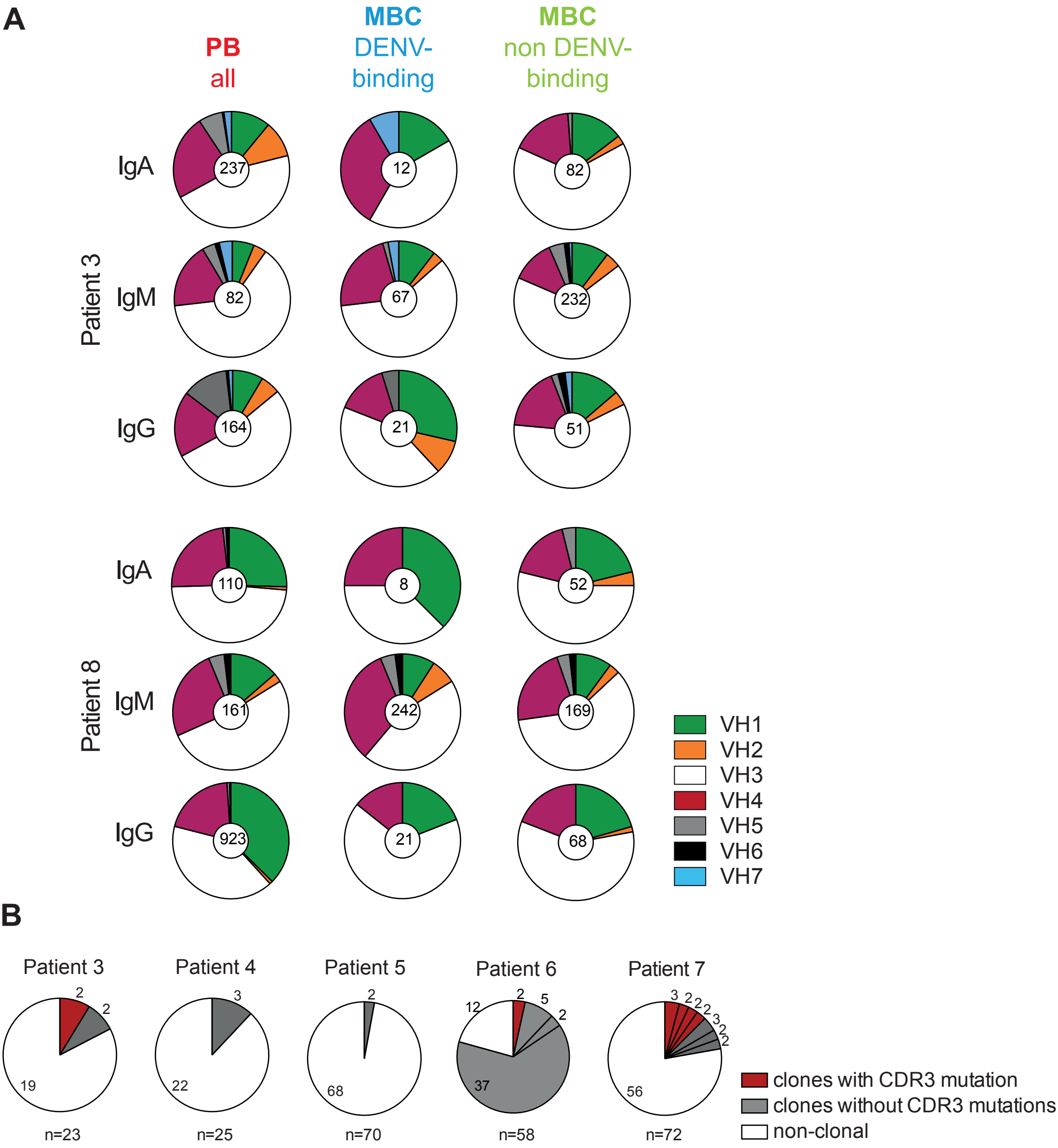

Supplementary Figure 2: **Isotype-independent VH bias and limited clonal expansion in PBs.** A) VH usage for IgG⁺, IgM⁺ and IgA⁺ sequences in patients 3 and 8 as determined by 454 sequencing. Only unique CDR3 sequences were included in the analysis. B) Clonal expansion amongst sequences from single sorted PBs. “Clones without CDR3 mutations” were defined as CDR3 sequences with identical V, D and J gene usage, the same length and identical amino acid sequenes. CDR3 sequences in “Clones with CDR3 mutation” used the same V,D and J gene and had the same lenght but differed by at least one amino acid. The number of CDR3 sequence for clones (red and grey areas) and for non-related CDR3 sequences is indicated inside or next to the charts. The total number of single sequences analyzed is mentioned below the pie charts. Limited clonal expansion for patients 1 and 2 were reported previously (Xu M, Hadinoto V et al., J Immunol 2012, 189(12): 5877-5885)

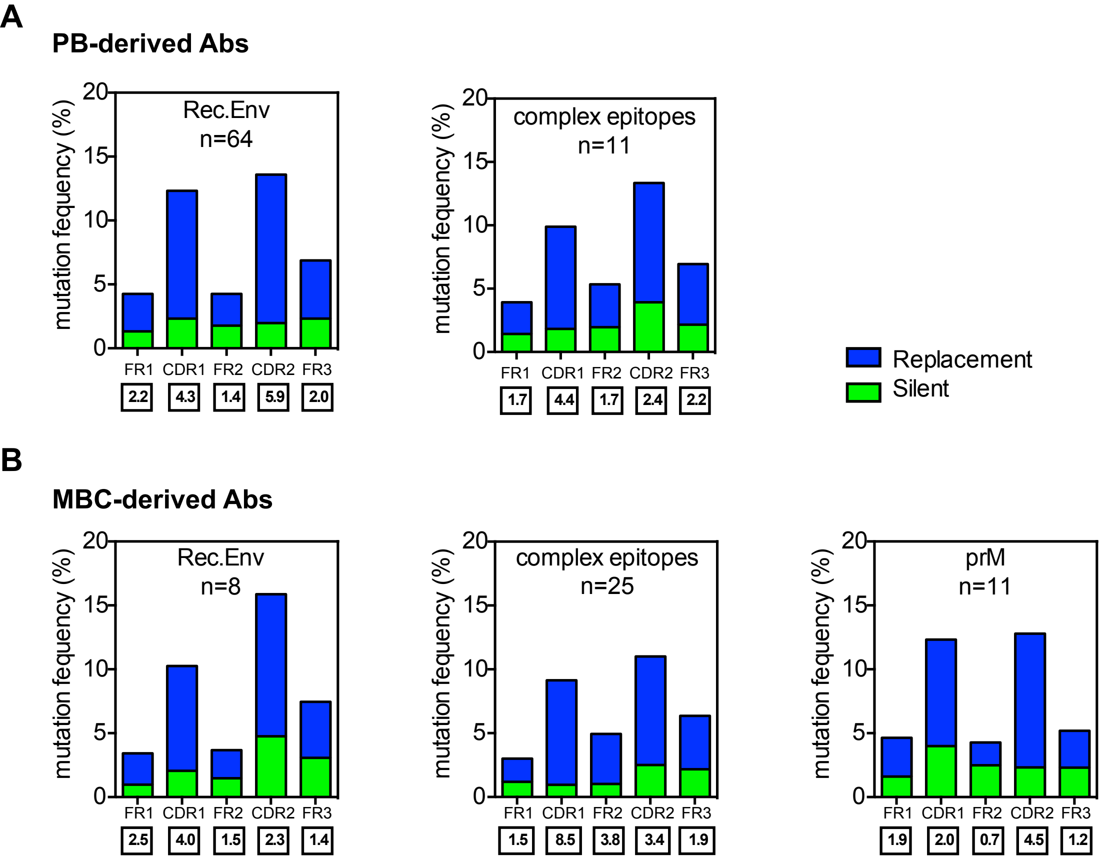

Supplementary Figure 3: **Plasmablasts (PB) and memory B cells (MBC) show similar mutation rates.** The frequency of replacement (R) versus silent (S) mutations in the VH region excluding CDR3 was calculated for every group of antibodies as indicated in the graph titels, both for plasmablast (PB)-derived Abs (A) and for MBC-derived Abs (B). The primer-binding region in FR1 was omitted for the analysis. The ratio of R/S mutations is indicated in boxes below each bar. The number n of Ab sequences analyzed for each graph is indicated.
