# Supplementary material for: Plasmablasts During Acute Dengue Infection Represent a Small Subset of a Broader Virus-specific Memory B Cell Pool
Source: eBioMedicine. 2016 Sep 7;12:178–88. doi: 10.1016/j.ebiom.2016.09.003 (PMC5078588; doi:10.1016/j.ebiom.2016.09.003)
Supplement: Supplementary file 2 — Supplementary tables 1-3. [file mmc2.pdf]

Primers	Sequence	Use
VH1_7-1	ATGGACTGSAYYTGGAGGATCCTC	RT-PCR
VH1-2	ATGGACTGGACCTGGAGVRTCYT	RT-PCR
VH2-1	ATGGACACACTTTGCTMCACRCTC	RT-PCR
VH3-1	ATGGAAYTGGGGCTSMGCTGGGTT	RT-PCR
VH3-2	ATGGAGTTTGGGCTGAGCTGGVTT	RT-PCR
VH3-3	ATGGAGTTGGGRCTGWGCTGGRTT	RT-PCR
VH3-4	AYGGAGTTTKGRCTKAGCTGGGTT	RT-PCR
VH4-1	ATGAARCAYCTGTGGTTYTTCCT	RT-PCR
VH5-1	ATGGGGTCAACCGCCATCCTYGSC	RT-PCR
VH6-1	ATGTCTGTCTCCTTCCTCATCTTCC	RT-PCR
VK1-1	ATGGACATGAGRGTCCYYGCTCAGC	RT-PCR
VK1-2	ATRGACATGAGGGTSCCCGCTCAGC	RT-PCR
VK2-1	ATGAGGCTCCYTGCTCAGCTYCTG	RT-PCR
VK3-1	ATGGAARCCCCAGCKCAGCTTCTC	RT-PCR
VK3-2	ATGGAACCATGGAAGCCCCAGCAC	RT-PCR
VK4-1	ATGGTGTTGCAGACCCAGGTCTTC	RT-PCR
VK5-1	ATG GGG TCC CAG GTT CAC CTC CTC	RT-PCR
VK6-1	ATGTTGCCATCACAACTCATTGGG	RT-PCR
VK6-2	ATGGTGTCCCCGTTGCAATTCCTG	RT-PCR
VL1-1	ATGGCCTGGTCYCCTCTCYTCCTC	RT-PCR
VL1-2	ATGRCCDGCTYCCCTCTCCTCCTC	RT-PCR
VL2	ATGGCCTGGGCTCTGCTSCTCCTC	RT-PCR
VL3-r	ATGGCATGGATCCCTCTCTTCCTC	RT-PCR
VL3-j	ATGGCCTGGACCGCTCTCCTTCTG	RT-PCR
VL3-pa19	ATGGCCTGGAYCCCTCTCCTGCTC	RT-PCR
VL3-l	ATGGCCTGGACCCCTCTCTGGCTC	RT-PCR
VL3-h	ATGGCCTGGACCGTTCTCCTCCTC	RT-PCR
VL3-e	ATGGCATGGGCCACACTCCTGCTC	RT-PCR
VL3-m	ATGGCCTGGATCCCTCTACTTCTC	RT-PCR
VL4-c	ATGGCCTGGGTCTCCTTCTACCTA	RT-PCR
VL4-a	ATGGCCTGGACCCAACTCCTCCTC	RT-PCR
VL4-b	ATGGCTTGGACCCCACTCCTCTTC	RT-PCR
VL5-1	ATGGCCTGGACTCYCTCTYCTYCTC	RT-PCR
VL6	ATGGCCTGGGCTCCACTACTTCTC	RT-PCR
VL7	ATGGCCTGGACTCCTCTCTTTCTG	RT-PCR
VL8	ATGGCCTGGATGATGCTTCTCCTC	RT-PCR
VL9	ATGGCCTGGGCTCCTCTGCTCCTC	RT-PCR
VL10	ATGCCCTGGGCTCTGCTCCTCCTG	RT-PCR
IgG 227-reverse	GTCCACCTTGGTGTTGCTGGGCTT	RT-PCR
IgM-reverse	TGGAAGAGGCACGTTCTTTTCTTT	RT-PCR
HuCκ-3'-reverse	ACACTCTCCCCTGTTGAAGCTCTT	RT-PCR
HuCλ2-3'reverse	TGAACATTCTGTAGGGGCCACTG	RT-PCR
HuCλ7-3'reverse	AGAGCATTCTGCAGGGGCCACTG	RT-PCR

Primers	Sequence	Use
HuVH1B/7A-BACK-Sal I	GGGTTCCAGGGTCGACtggcCAGRTGCAGCTGGTGCARTCTGG	Cloning PCR
HuVH1C-BACK-Sal I	GGGTTCCAGGGTCGACtggcSAGGTCCAGCTGGTRCAGTCTGG	Cloning PCR
HuVH2B-BACK-Sal I	GGGTTCCAGGGTCGACtggcCAGRTCACCTTGAAGGAGTCTGG	Cloning PCR
HuVH3B-BACK-Sal I	GGGTTCCAGGGTCGACtggcSAGGTGCAGCTGGTGGAGTCTGG	Cloning PCR
HuVH3C-BACK-Sal I	GGGTTCCAGGGTCGACtggcGAGGTGCAGCTGGTGGAGWCYGG	Cloning PCR
HuVH4B-BACK-Sal I	GGGTTCCAGGGTCGACtggcCAGGTGCAGCTACAGCAGTGGGG	Cloning PCR
HuVH4C-BACK-Sal I	GGGTTCCAGGGTCGACtggcCAGSTGCAGCTGCAGGAGTCSGG	Cloning PCR
HuVH5B-BACK-Sal I	GGGTTCCAGGGTCGACtggcGARGTGCAGCTGGTGCAGTCTGG	Cloning PCR
HuVH6A-BACK-Sal I	GGGTTCCAGGGTCGACtggcCAGGTACAGCTGCAGCAGTCAGG	Cloning PCR
HuVk1B-BACK-Sal I	GGGTTCCAGGGTCGACtggcGACATCCAGWTGACCCAGTCTCC	Cloning PCR
HuVk2-BACK-Sal I	GGGTTCCAGGGTCGACtggcGATGTTGTGATGACTCAGTCTCC	Cloning PCR
HuVk3B-BACK-Sal I	GGGTTCCAGGGTCGACtggcGAAATTGTGWTGACRCAGTCTCC	Cloning PCR
HuVk4B-BACK-Sal I	GGGTTCCAGGGTCGACtggcGATATTGTGATGACCCACACTCC	Cloning PCR
HuVk5-BACK-Sal I	GGGTTCCAGGGTCGACtggcGAAACGACACTCACGCAGTCTCC	Cloning PCR
HuVk6-BACK-Sal I	GGGTTCCAGGGTCGACtggcGAAATTGTGCTGACTCAGTCTCC	Cloning PCR
HuVL1A-BACK-Sal I	GGGTTCCAGGGTCGACtggcCAGTCTGTG CTGACTCAGCCACC	Cloning PCR
HuVL1B-BACK-Sal I	GGGTTCCAGGGTCGACtggcCAGTCTGTGYTGACGCAGCCGCC	Cloning PCR
HuVL1C-BACK-Sal I	GGGTTCCAGGGTCGACtggcCAGTCTGTCGTGACGCAG CCG CC	Cloning PCR
HuVL2-BACK-Sal I	GGGTTCCAGGGTCGAC tggcCARTCTGCCCTGACTCAGCCT	Cloning PCR
HuVL3A-BACK-Sal I	GGGTTCCAGGGTCGACtggcTCCTATGWGCTG ACTCAG CCACC	Cloning PCR
HuVL3B-BACK-Sal I	GGGTTCCAGGGTCGAC tggcTCTTCT GAGCTGACTCAGGACCC	Cloning PCR
HuVL4-BACK-Sal I	GGGTTCCAGGGTCGACtggcCACGTT ATACTGACTCAACCG CC	Cloning PCR
HuVL5-BACK-Sal I	GGGTTCCAGGGTCGACtggcCAGGCT GTGCTGACTCAGCCGTC	Cloning PCR
HuVL6-BACK-Sal I	GGGTTCCAGGGTCGAC tggcAATTTT ATGCTGACTCAGCCCCA	Cloning PCR

HuVL7/8-BACK-Sal I	GGGTTCCAGGGTCGACtggcCAG RCTGTGGTGACYCAGGAGCC	Cloning PCR
HuVL9-BACK-Sal I	GGGTTCCAGGGTCGACtggcCWG CCTGTGCTGACT CAGCCMCC	Cloning PCR
HuIgG-NheI reverse+4	TCTTGGAGGAGGGTGCTAGCGGGAAGACCGATGGGCCCT	Cloning PCR
Hu kappa BbvCI reverse	TCAGGGTGCTGCTGAGGCTGTAG	Cloning PCR
HuIgL2-Not I reverse	CGGCCTCGAGCGGCCGCTTATGAACATTCTGTAGGGGCCA	Cloning PCR
HuIgL7-Not I reverse	CGGCCTCGAGCGGCCGCTTAAGAGCATTCTGCAGGGGCCA	Cloning PCR

Supplementary Table S1: Primers used for sequencing and cloning of single B cells

Patient number	Patient ID	Infection	Current serotype of infection	plasmablasts sorted as single cells/for 454 analysis	plasmablast sequences from single cell/454 analysis*	DENV-specific plasmablast antibodies (total cloned mAbs)	DENV-2 MBCs sorted as single cells/ for 454 sequencing	DENV-2 MBC sequences from single cell/454 analysis*	Specific DENV-2 MBC antibodies (total cloned mAbs)	DENV-3 MBCs sorted as single cells/ for 454 sequencing	DENV-3 MBC sequences from single cell/454 analysis*	Specific DENV-3 MBC antibodies (total cloned mAbs)	neg. MBCs sorted as single cells/ for 454 sequencing	neg. MBCs sequences from single cell/454 analysis*
1	10/63	secondary	DENV-2	single cells	117	23 (31)	single cells	66	20 (36)	-	-	-	single cells	39
2	10/50	secondary	DENV-3	single cells	94	19 (30)	single cells	89	9 (15)	single cells	64	7 (21)	single cells	47
3	E1392	secondary	DENV-2	single cells/ 4,720	23/48,646	6 (13)	-	-	-	single cells/7,078	24/28,201	7 (10)	34,547	34,156
4	E1183	secondary	DENV-2	single cells	25	2 (6)	-	-	-	single cells	28	2 (5)	single cells	13
5	E1407	secondary	DENV-2	single cells	70	16 (20)	-	-	-	-	-	-	single cells	29
6	E1311	secondary	DENV-2	single cells	58	7 (15)	-	-	-	-	-	-	-	-
7	C07	secondary	DENV-2	single cells	72	14 (22)	-	-	-	-	-	-	-	-
8	E1291	secondary	DENV-2	12,301	41,400	-	-	-	-	5,499	30,694	-	45,419	21,796
9	E1385	primary	DENV-2	single cells	69	1 (13)	-	-	-	-	-	-	-	-
10	C01	primary	DENV-2	single cells	49	2 (7)	-	-	-	-	-	-	-	-
11	E1414	primary	DENV-2	1,065	21,982	-	4,216	31,816	-	-	-	-	12,524	29,385
12	E1465	primary	DENV-2	11,830	20,062	-	648	18,108	-	-	-	-	12,004	25,643

Supplementary Table S2: Summary of sorted cells, number of sequenced cells, number of sequences and number of antibodies expressed per patient. Cell numbers are based on the FACS sorter counting.
*) total mapped reads are given for 454 sequencing analysis

Patient number	Patient ID	Infection	Current serotype of infection	Frequency of CD27+CD38++/ total lymphocytes	Frequency of CD27+MBC-DENV-2 binding/ CD19+CD20+ (%)	Frequency of CD27+MBC-DENV-3 binding/ CD19+CD20+ (%)
1	10/63	secondary	DENV-2	3.0	1.4 (sorted for CD20+, DENV-2+ cells gated from CD19+CD27+)	-
2	10/50	secondary	DENV-3	19	1.0	8.1
3	E1392	secondary	DENV-2	0.9	-	5.4
4	E1183	secondary	DENV-2	0.2	-	0.5
5	E1407	secondary	DENV-2	1.0	-	-
6	E1311	secondary	DENV-2	6.0	-	-
7	C07	secondary	DENV-2	0.9	-	-
8	E1291	secondary	DENV-2	1.7	-	1.9
9	E1385	primary	DENV-2	0.1	-	-
10	C01	primary	DENV-2	0.6	-	-
11	E1414	primary	DENV-2	0.1	2.5	-
12	E1465	primary	DENV-2	0.6	0.7	-

Supplementary Table S3: Numbers of CD27++CD38++ plasmablasts and numbers of DENV-binding memory B cells in patient sampels used for cell sorting in this study.
